# Differential Transcriptome Analysis between *Paulownia fortunei* and Its Synthesized Autopolyploid

**DOI:** 10.3390/ijms15035079

**Published:** 2014-03-21

**Authors:** Xiaoshen Zhang, Minjie Deng, Guoqiang Fan

**Affiliations:** Institute of Paulownia, Henan Agricultural University, 95 Wenhua Road, Jinsui District, Zhengzhou 450002, Henan, China; E-Mails: abf1232@163.com (X.Z.); dengmj1980@126.com (M.D.)

**Keywords:** *Paulownia fortunei*, transcriptome, polyploidy, *de novo* assembly, next-generation sequencing

## Abstract

*Paulownia fortunei* is an ecologically and economically important tree species that is widely used as timber and chemical pulp. Its autotetraploid, which carries a number of valuable traits, was successfully induced with colchicine. To identify differences in gene expression between *P. fortunei* and its synthesized autotetraploid, we performed transcriptome sequencing using an Illumina Genome Analyzer IIx (GAIIx). About 94.8 million reads were generated and assembled into 383,056 transcripts, including 18,984 transcripts with a complete open reading frame. A conducted Basic Local Alignment Search Tool (BLAST) search indicated that 16,004 complete transcripts had significant hits in the National Center for Biotechnology Information (NCBI) non-redundant database. The complete transcripts were given functional assignments using three public protein databases. One thousand one hundred fifty eight differentially expressed complete transcripts were screened through a digital abundance analysis, including transcripts involved in energy metabolism and epigenetic regulation. Finally, the expression levels of several transcripts were confirmed by quantitative real-time PCR. Our results suggested that polyploidization caused epigenetic-related changes, which subsequently resulted in gene expression variation between diploid and autotetraploid *P. fortunei*. This might be the main mechanism affected by the polyploidization. Our results represent an extensive survey of the *P. fortunei* transcriptome and will facilitate subsequent functional genomics research in *P. fortunei*. Moreover, the gene expression profiles of *P. fortunei* and its autopolyploid will provide a valuable resource for the study of polyploidization.

## 1. Introduction

*Paulownia* is a genus native to China. The two species most often cultivated in China are *Paulownia fortunei* and *Paulownia elongata. Paulownia* is a fast-growing, short-rotation timber crop that is also valuable for the production of chemical pulp [1,2]. Additionally, its wood exhibits rot resistance, dimensional stability and a high ignition point; thus, *Paulownia* is widely used for making furniture, aircraft, plywood, toys and musical instruments [3]. The tolerance of *Paulownia* to drought and soil extremes makes it commercially important for use in the reclamation of surface-mined land [4]. Indeed, this genus has been suggested for the reforestation of nutrient-poor soils [5].

Polyploidy is the heritable condition of possessing more than two complete sets of chromosomes [6]. Generally, polyploids are divided into autopolyploids arising by chromosome doubling of a single species and allopolyploids arising via interspecific hybridization and subsequent chromosome doubling. They play an important role in plant evolution. Recent estimates indicate that 15% of angiosperm and 31% of fern speciation events are accompanied by an increase in ploidy [7]. Many major crops, including wheat, cotton, oat, coffee, potato and oilseed rape, are polyploids [8,9]. Polyploids have also been induced by experimental treatments, such as heat shock and colchicines [10]. Rapid genomic and gene expression changes have been demonstrated in many synthesized and natural polyploid plants, including *Arabidopsis* [11,12], *Brassica* [13,14], *Gossypium* [15], *Spartina* [16] and *Tragopogon* [17].

Polyploidy provides genome “buffering” by increasing allelic diversity and heterosis, and it facilitates the creation of novel phenotypic variation and asexual reproduction [6,18], which may be valuable in plant breeding. With the goals of enriching the germplasm of *Paulownia* and increasing the desired traits, our lab successfully induced an autopolyploid of *P. fortunei* with the desirable wood properties using colchicine [19]. We were unable to characterize the genetic differences between diploid and autotetraploid *P. fortunei* on a grand scale, due to a lack of genomic sequence data, until the development of high-throughput next-generation sequencing (NGS). This technology allowed us to perform a short read-based transcriptome analysis of non-model organisms for whom the genomic sequence is unknown [20]. In the present study, we sequenced the transcriptome of diploid and autotetraploid *P. fortunei* using the Illumina Genome Analyzer IIx (GAIIx), an NGS platform, assembled and annotated the sequence data and analyzed the gene expression changes caused by polyploidization. These assembled transcriptome sequences and annotations will be useful for future functional genomic analyses.

## 2. Results

### 2.1. Illumina Paired-End Sequencing and De Novo Assembly

**Figure 1.**
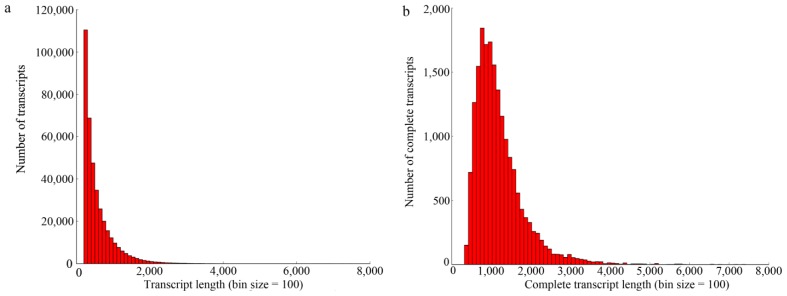
Overview of *P. fortunei* transcriptome assembly. (**a**) The size distribution of the transcripts obtained from *de novo* assembly of high-quality clean reads; (**b**) the size distribution of the transcripts with a complete open reading frame (ORF).

The two cDNA libraries, which were respectively constructed using the leaves of diploid *P. fortunei* (PF_2_) and its autotetraploid (PF_4_), were sequenced using the Illumina GAIIx sequencing platform. We obtained a total of 96.4 million 100-bp raw reads from the two libraries (51.2 million for PF_2_; 45.2 million for PF_4_), encompassing a total of 10.4 Gbp. After a stringent quality assessment and data filtering, about 94.8 million high-quality reads of both ploidies with a base quality score >20 were deposited in the National Center for Biotechnology Information (NCBI) Short Read Archive (accession number: SRP032166) and used to assemble the transcriptome of *P. fortunei* with the Trinity program [21]. The complete read dataset was assembled into 383,056 transcripts totaling 218,296,008 bp. The size of the transcripts ranged from 201 to 10,538 bp, with a mean length of 570 bp (Figure 1a). Furthermore, a total of 18,984 assembled transcripts with a complete open reading frame (ORF) and their corresponding protein sequences were predicted using TransDecoder in the Trinity package. The length of the complete transcripts with a total of 22,724,258 bp varied from 319 to 7406 bp, with an average of 1197 bp (Figure 1b).

### 2.2. Annotation of the Predicted Complete Transcripts

**Figure 2.**
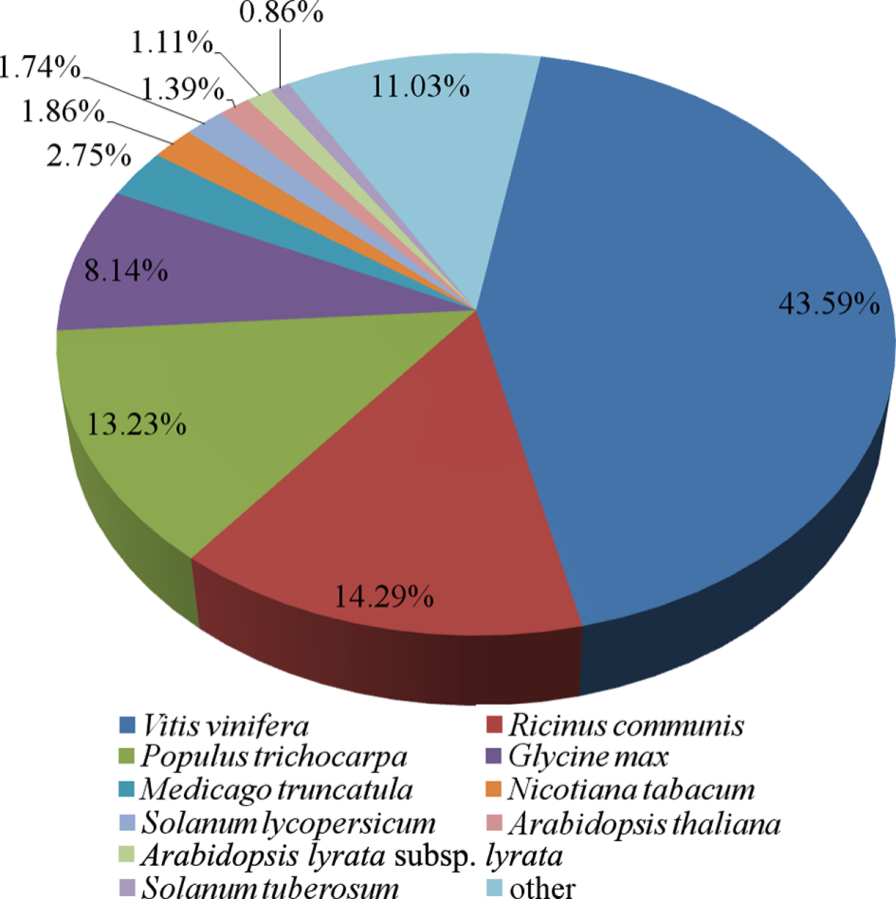
Species distribution of the standard protein-protein BLAST (BLASTp) matches of *P. fortunei* transcripts against the non-redundant (nr) database (*E*-value cut-off 1.0 × 10^−5^).

We aligned 18,984 protein sequences corresponding to the ORFs of the complete transcripts with sequences from public protein databases using the BLASTp program (*E*-value cut-off: 1.0 × 10^−5^). The results showed that 16,004 (84.3%), 10,730 (56.5%), 10,100 (53.2%), 4697 (24.7%) and 5076 (26.7%) transcripts had homologous sequences in the NCBI non-redundant (nr) (ftp://ftp.ncbi.nih.gov/blast/db/FASTA/nr.gz), UniProtKB/Swiss-Prot (http://www.uniprot.org/), Eukaryotic Orthologous Groups (KOG) (http://www.ncbi.nlm.nih.gov/COG/), Pfam (http://pfam.sanger.ac.uk/) and Kyoto Encyclopedia of Genes and Genomes (KEGG) (http://www.genome.jp/kegg/) databases, respectively. For the nr annotations, most transcripts had significant similarity to sequences from *Vitis vinifera* (6976; 43.6%), *Ricinus communis* (2287; 14.3%) and *Populus trichocarpa* (2118; 13.2%) (Figure 2).

### 2.3. Functional Classification Using GO, KOG and KEGG

First, the proteins corresponding to complete transcripts were annotated using the Pfam database. Using the gene ontology (GO) terms associated with the Pfam annotations, we classified 4697 transcripts into 33 functional groups under three main divisions: biological processes, cellular components and molecular functions (Figure S1). In the molecular function category, a significant percentage of transcripts were assigned to “binding” (2332; 51.7%) and “catalytic activity” (1784; 39.6%). In the cellular components category, a high percentage of transcripts were assigned to “cell” (566; 26.8%), whereas many transcripts were assigned to “metabolic processes” (1950; 42.4%) and “cellular processes” (1574; 34.2%) for the functional class biological processes.

After alignment to the KOG database, the functions of 10,100 transcripts were assigned to 24 categories (Figure S2). “Posttranslational modification, protein turnover and chaperones” represented the largest group (1097; 10.8%). However, categories with no concrete assignment, such as “function unknown” (598; 5.9%) and “general function prediction only” (2072; 20.5%) accounted for a large fraction of the transcripts.

To reconstruct the metabolic pathways in *P. fortunei*, 5076 transcripts having enzyme commission numbers were assigned to 235 KEGG pathways (Table S1). In terms of metabolism, the greatest numbers of transcripts were matched to “metabolic pathways” (1413; 27.8%), “biosynthesis of secondary metabolites” (735; 14.5%) and “microbial metabolism in diverse environments” (394; 7.8%). In the KEGG database, the top five pathways, including the most transcripts, were “RNA transport” (185; 3.6%), “protein processing in endoplasmic reticulum” (159; 3.1%), “spliceosome” (157; 3.1%), “glycolysis/gluconeogenesis” (156; 3.1%) and “starch and sucrose metabolism” (148; 2.9%).

### 2.4. Analysis of Differentially Expressed Transcripts between Diploid and Autotetraploid P. fortunei

A total of 1158 out of 18,984 (6.09%) complete transcripts were significantly differentially expressed between diploid and autotetraploid *P. fortunei*. Six hundred fifty eight were upregulated and 500 were downregulated in autotetraploid *P. fortunei* when compared with the diploid sample. For upregulated transcripts, differences ranged between 2.17- and 10.65-fold; for downregulated transcripts, differences ranged between 2.59–10.89-fold. Four hundred and eighty-three transcripts were only detected in the autotetraploid sample, and three hundred and seventy-eight transcripts were only detected in the diploid sample. A total of 983, 624, 317 and 317 transcripts were annotated in the NCBI nr, KOG, Pfam and KEGG databases, respectively.

### 2.5. Differentially Expressed Transcripts Related to Energy Metabolism

We mapped differentially expressed transcripts (DETs) to terms in the KEGG database and compared them with the whole transcriptome, with a focus on finding genes involved in metabolic pathways that were significantly enriched. Up to 16 KEGG pathways were significantly enriched (Table 1), with “pyruvate metabolism” (map00620), “carbon fixation in photosynthetic organisms” (map00710) and “oxidative phosphorylation” (map00190) as the top three pathways. Notably, these three pathways, “sulfur metabolism” (map00920) and “photosynthesis-antenna proteins” (map00196) are parts of energy metabolism. In the pathway “oxidative phosphorylation”, four transcripts corresponding to four V-type (vacuolar or vesicular proton pump) H^+^-transporting ATPase subunits (K02155, K02147, K02154 and K02145) were upregulated. Seven upregulated and two downregulated transcripts corresponding to five enzymes (K00025, K01006, K00873, K00029 and K01595) were involved in the pathway “carbon fixation in photosynthetic organisms”; while these five enzymes also play roles in the pathway “pyruvate metabolism” belonging to carbohydrate metabolism (Table 2). Two transcripts, m.50116 and m.50118, also were involved in the pathway “carbon fixation pathways in prokaryotes” (map00720).

### 2.6. Transcriptomic Changes Related to Genetic Information Storage and Processing

In the KOG database, one hundred and thirty-five DETs were classified to the main category “information storage and processing”, represented by five functional classes (Figure S3 and Table S2). The category containing the most number of DETs (49) was “RNA processing and modification”, including splicing factor (10), RNA helicase (12), RNA methylase (three), the subunit of mRNA cleavage and polyadenylation factor (four). Thirty-seven DETs were assigned to the category “Translation, ribosomal structure and biogenesis”; 14 were upregulated, and 23 were downregulated. Fifteen differentially expressed transcription factors (three upregulated GATA transcription factors) and two coactivators were included in the category “transcription”. Twelve upregulated and five downregulated DETs, such as five-fold upregulated mismatch repair ATPase MSH6 (m.22798) and four-fold exonuclease HKE1/RAT1 (m.56286), were divided into the category “replication, recombination and repair”. Eight DETs (six upregulated and two downregulated) were allocated to the category “chromatin structure and dynamics”, for example, the subunit CPS60/ASH2/BRE2 of the histone H3 (Lys4) methyltransferase complex and a component, SWI2, of chromatin remodeling complex SWI/SNF. These results suggested the transmission pipeline of genetic information might change during the shift from di- to tetra-ploid.

### 2.7. Verification of DETs by Quantitative Real-Time PCR

**Figure 3.**
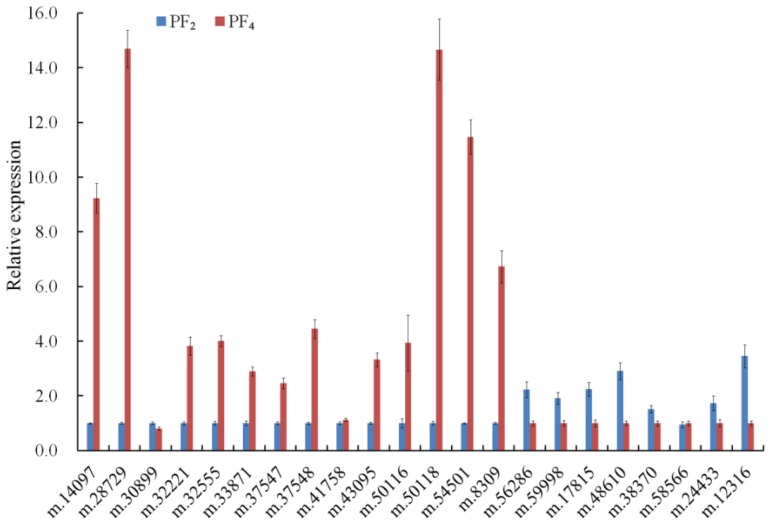
Quantitative real-time PCR (qRT-PCR) analysis of differentially expressed transcripts involved in energy metabolism. PF_2_, diploid *P. fortunei*; PF_4_, autotetraploid *P. fortunei*. Bars represent the mean (±SD).

Twenty-two DETs were selected for quantitative real-time PCR (qRT-PCR) verification with specific primers (Tables 2, 3 and S3). Twelve transcripts, including two (m.8309 and m.32221) that were downregulated in PT4 *vs*. PT2, expressed at a higher level, and seven transcripts expressed at a lower level in autotetraploid plants than that in diploid plants; whereas there was almost no difference in the expression of the other three transcripts in diploid and autotetraploid *P. fortunei* (Figure 3). Twelve upregulated transcripts indicated that the energy and carbohydrate metabolism level of autotetraploid *P. fortunei* was probably higher than its diploid progenitor. Eight upregulated transcripts related to carbon fixation in autotetraploid plants were confirmed, which help us to understand our previous report that the wood density and fiber length of autotetraploid *P. fortunei* increased compared with its diploid progenitor [22]. Seven downregulated transcripts confirmed the variation of chromatin remodeling, the mRNA process and transcript regulation during the polyploidization of *P. fortunei*. In addition, for seventeen of twenty-two transcripts, their expression change trends (up- or down-regulation) determined by the qRT-PCR were in agreement with those predicted by the bioinformatic tool, which suggested that our transcriptome data were reliable.

## 3. Discussion

In the area of genomics research, NGS technology offers higher throughput and a lower cost than Sanger sequencing. The Illumina GAIIx (Illumina Inc., San Diego, CA, USA), Roche/454 Genome Sequencer (Roche Diagnostics Corp., Basel, Switzerland) and ABI SOLiD System (Life Technologies Corp., Carlsbad, NM, USA) are the three most widely used NGS platforms for genome sequencing, genome resequencing, transcriptome sequencing, miRNA expression profiling and DNA methylation analysis [23,24]. NGS technology can also be used for *de novo* transcriptome sequencing of non-model organisms, thereby facilitating the study of organisms with an unknown reference genome on a large scale [20]. In the present study, an Illumina GAIIx was used for *de novo* transcriptome sequencing of *P. fortunei*, because of its low cost and ability to generate large numbers of reads. About 94.8 million high-quality reads were generated and assembled *de novo* to 383,056 transcripts, including 18,984 transcripts with a complete ORF. Compared with the *de novo* transcriptome of *P. tomentosa* × *P. fortunei* in our previous work, in our present study, the number of complete transcripts was less, and the mean length of complete transcripts was shorter, which suggested that the total number of reads probably affected the assembly quality [25]. A total of 16,004 complete transcripts were successfully annotated using the NCBI nr database, suggesting that their functions were relatively conserved.

Most plant transcriptome studies have assembled sequence data from different tissues [26–28] or used mixed cDNAs from different tissues as a sequencing sample [29–31]. In this case, transcriptomic data could be acquired, but since alternative splicing exists in different tissue types [32], assembly was difficult. A few experiments have used tissue-specific transcriptomic sequencing and assembly [33,34], which can produce more accurate data than the former strategy. Tissue-specific transcriptomic data will supply a good reference set for gene expression studies, especially in non-model plants.

Full coding sequence cDNAs are useful in functional studies of genes and gene products and in genome assembly [35]. Though the prediction of coding sequence regions in eukaryotic genomes is complicated by the interruption of introns and the low proportion of protein coding sequences in the genome, previous studies in other species have produced full-length cDNA sets [36,37]. Up to now, few full-length cDNAs were available in public databases for *Paulownia*. We herein attempted to identify transcripts with full-length cDNA sequences from the reads using the Trinity program, with the aim of providing a reference for the future identification of coding sequences in the lab. In addition, we selected transcripts with an ORF as representatives for differentially expressed gene profiling between diploid and autotetraploid *P. fortunei* to decrease the inference of non-coding sequences and the occurrence of false-positive results.

The molecular basis of plant polyploids is probably correlated with genomic sequence and gene expression changes. Large-scale gene expression changes that resulted from the combination of hybridization and genome doubling were observed in allopolyploids [38]; meanwhile, there was only a small percentage of transcriptome alteration in autotetraploids [39], even no differences [40,41], compared with their diploid progenitors. A low level (6.09%) of gene expression alteration between *P. fortunei* diploid and autotetraploid is similar to the transcriptome data previously reported for *Arabidopsis*, *Isatis indigotica*, *Eragrostis curvula* and *Siraitia grosvenorii* [42–45]. The changes of genes related to metabolism process were significant between *S. grosvenorii* diploid and tetraploid [45]. In our study, differentially expressed transcripts related to energy metabolism and carbon fixation were enriched; most of them were upregulated.

Recent studies of polyploid plants have shown that genome-wide changes in gene expression may be associated with the inter-related epigenetic mechanisms (DNA methylation with histone covalent modifications and small RNAs) [46]. Salmon *et al*. [16] suggested that significant changes of DNA methylation patterns could explain the morphological plasticity and larger ecological amplitude of *Spartina* allopolyploids. Transcriptome alterations of *A. thaliana* autotetraploid Col-0 lines were related to DNA methylation, which worked with other DNA modifications [11]. Several siRNAs correlated with repeat sequences or transposons in *A. suecica* varied significantly between the two progenitor species, *A. thaliana* and *A. arenosa* [47]. In our results, the predicted functions of some DETs were connected to DNA or histone methyl transfer, RNA processing and chromosome remodeling; this indirectly indicated epigenetic mechanisms altered by polyploidization. On one hand, epigenetic changes may alter the expression of one gene; on the other hand, these changes acting on one transcription factor/repressor could alter the expression of a number of target and downstream genes without any further change of their epigenetic state, which caused the differential transmission of genetic information, as well as physiological, biochemical and phenotypic variation between diploid and tetraploid plants.

## 4. Experimental Section

### 4.1. Tissue Collection and RNA Isolation

Leaves were respectively collected from twenty (10 for PF_2_; 10 for PF_4_) healthy tissue culture seedlings grown for 30 days. All seedlings were incubated at 25 ± 2 °C under a 16-h/8-h light/dark photoperiod with light supplied by cool-white fluorescent lamps at an intensity of 130 μmol·m^−2^·s^−1^. Equal amounts of the leaves from ten diploid (or autopolyploid) seedlings were mixed as one sample. Total RNA was respectively isolated from two mixed leaf samples using a Plant RNA Isolation Kit (AutoLab, Beijing, China), followed by RNA purification using an RNeasy MiniElute Cleanup Kit (Qiagen, Valencia, CA, USA), according to the manufacturer’s protocol.

### 4.2. cDNA Library Preparation, Sequencing and De Novo Assembly

Two paired-end libraries were constructed using a TruSeq RNA Sample Preparation Kit (Illumina, San Diego, CA, USA), according to the manufacturer’s instructions. The high-throughput sequencing was conducted using an Illumina GAIIx platform. A primary analysis of the data and base calling were performed using the software built into the Illumina instrument.

The raw image data were transformed by base calling into sequence data, which were called raw reads. Before transcriptome assembly, a stringent raw reads filtering process using the software package SolexaQA (DynamicTrim.pl, *p* = 0.05; LengthSort.pl, min length = 25) was employed to acquire clean reads [48]. Reads in which >10% of the bases had a quality score of *Q* < 20, ambiguous sequences represented as “N” and adaptor contamination were removed. We used the Trinity program (version: trinityrnaseq-r2013-02-25, the options: -seqType fq -min_contig_length 200 -group_pairs_distance 250 -min_kmer_cov 2) to *de novo* assemble the clean reads [21]. TransDecoder in the Trinity package (the options: -m 100 -G universal -C complete ORFs only -T 500) was used to predict the open reading frames (ORFs) of the assembled transcripts and their corresponding protein sequences.

### 4.3. Functional Annotation and Categorization of the Transcripts

Protein sequences corresponding to the ORFs of the complete transcripts were subjected to a similarity search against several public databases, including the NCBI non-redundant protein sequence database (nr) (ftp://ftp.ncbi.nih.gov/blast/db/FASTA/nr.gz), UniProtKB/Swiss-Prot (http://www.uniprot.org/), Eukaryotic Orthologous Groups (KOG) (http://www.ncbi.nlm.nih.gov/COG/), the Kyoto Encyclopedia of Genes and Genomes (KEGG) (http://www.genome.jp/kegg/) [49] and Pfam (http://pfam.sanger.ac.uk/), using BLASTp (NCBI, Bethesda, MD, USA) (version: 2.2.22, the options: -F F -e 1e-5 -p BLASTp) [50]. The complete transcripts aligned to the KOG database (http://www.ncbi.nlm.nih.gov/COG/) were classified according to their possible functions. The resulting hits from the Pfam database (http://pfam.sanger.ac.uk/) were processed by Blast2GO software (Instituto Valenciano de Investigaciones Agrarias, Moncada, Spain) to obtain gene ontology (GO) annotations of the complete transcripts [51], and then, WEGO software (Zhejiang University, Hangzhou, China) was used to perform GO functional classifications [52,53]. To summarize the pathways in *P. fortunei*, we mapped the annotated sequences to all pathways in the KEGG database (http://www.genome.jp/kegg/).

### 4.4. Expression Abundance Analysis

To analyze the expression levels of the complete transcripts, we first used the Bowtie aligner (version: 0.12.7, the options used are in the Supplementary information) to align the reads from *P. fortunei* and its synthesized autotetraploid back to the assembled reference transcriptome [54]. We then used RSEM (version: 1.2.2, the options used are in the Supplementary information) built in the Trinity package to compute fragments per kilobase per million reads (FPKM) values [55] and applied RSEM-coupled EBseq (version: 1.1.5) (University of Wisconsin, Madison, WI, USA) to calculate transcript abundance differences between the two samples. The fold change for each transcript between the samples were computed as the ratio of the FPKM values. Transcripts with an absolute value of a log_2_ fold change >2 and a *p*-value <0.01 were regarded as significantly differentially expressed transcripts.

### 4.5. Functional Analysis of DETs

Those DETs with a complete ORF were classified based on their KOG annotations, while the transcripts were mapped to all pathways in the KEGG database (http://www.genome.jp/kegg/). KEGG pathway enrichment analyses for these transcripts were performed by conducting hypergeometric tests with the assembled reference transcriptome set as the background. For the enrichment analysis, all *p*-values were adjusted using the Bonferroni correction. A corrected *p*-value of <0.05 was selected as the threshold for determining the significant enrichment of the transcript sets.

### 4.6. Quantitative Real-Time PCR Analysis

Total RNA extracted from the leaves of diploid *P. fortunei* and its autotetraploids was reverse transcribed into single-stranded cDNA with an iScript cDNA Synthesis Kit (Bio-Rad, Hercules, CA, USA). The SsoFast EvaGreen Supermix (Bio-Rad, Hercules, CA, USA) was used for qRT-PCR, starting with 1 μL cDNA template in a standard 20-μL reaction. The qRT-PCR cycle was as follows: 95 °C for 2 min, 40 cycles of 95 °C for 15 s and annealing at 57 °C for 15 s. The reactions were performed on a CFX96™ Real-Time PCR Detection System (Bio-Rad, Hercules, CA, USA), according to the manufacturer’s instructions. Two independent biological replicates for each sample and three technical replicates of each biological replicate were performed. The relative expression levels were calculated using the delta-delta *C*t method with normalization to the internal control 18SrRNA.

## 5. Conclusions

The present study investigated the transcriptome profiles of *P. fortunei* and its synthesized autopolyploid in an attempt to identify alterations in gene expression between them. The *de novo* characterization of the *P. fortunei* transcriptome will provide valuable information for functional genomics studies of *P. fortunei*, especially for the discovery of functional genes and protein expression. The detection of 1158 differentially expressed transcripts demonstrated that the gene expression changed after autopolyploidization, which would certainly facilitate further research into genetic and epigenetic mechanisms of *P. fortunei* polyploidization.

## Supplementary Information



## Figures and Tables

**Table 1.** Kyoto Encyclopedia of Genes and Genomes (KEGG) pathways significantly enriched for differentially expressed transcripts between diploid and tetraploid *P. fortune*.

Pathway entry	Pathway name	Number of DETs a	Corrected *p*-value
map00620	Pyruvate metabolism	20	0.008
map00710	Carbon fixation in photosynthetic organisms	17	0.011
map00190	Oxidative phosphorylation	11	0.023
map00720	Carbon fixation pathways in prokaryotes	9	0.009
map00860	Porphyrin and chlorophyll metabolism	9	0.009
map00906	Carotenoid biosynthesis	6	0.020
map00592	alpha-Linolenic acid metabolism	5	0.038
map00920	Sulfur metabolism	5	0.034
map00591	Linoleic acid metabolism	5	0.015
map00670	One carbon pool by folate	5	0.015
map00061	Fatty acid biosynthesis	4	0.021
map00590	Arachidonic acid metabolism	3	0.015
map00902	Monoterpenoid biosynthesis	3	0.012
map00196	Photosynthesis-antenna proteins	2	0.038
map00785	Lipoic acid metabolism	2	0.014
map00253	Tetracycline biosynthesis	2	0.016

a DETs means differentially expressed transcripts.

**Table 2.** KEGG annotations of 14 differentially expressed transcripts involved in the top three enriched metabolism pathways.

Transcript ID	KEGG orthology (KO) number	KEGG descriptions	*E*-value	KEGG pathway no.^a^
m.14097	K02155	V-type H^+^-transporting ATPase 16 kDa proteolipid subunit	7.0 × 10^−69^	map00190
m.54501	K02147	V-type H^+^-transporting ATPase subunit B	1.0 × 10^−45^	map00190
m.32555	K02154	V-type H^+^-transporting ATPase subunit I	1.0 × 10^−48^	map00190
m.33871	K02145	V-type H^+^-transporting ATPase subunit A	1.0 × 10^−48^	map00190
m.30899 *	K02144	V-type H^+^-transporting ATPase 54 kD subunit	7.0 × 10^−48^	map00190
m.8309 *	K00029	malate dehydrogenase (oxaloacetate-decarboxylating) (NADP^+^)	1.0 × 10^−34^	map00620, map00710
m.32221 *	K00029	malate dehydrogenase (oxaloacetate-decarboxylating) (NADP^+^)	8.0 × 10^−43^	map00620, map00710
m.28729	K00025	malate dehydrogenase	6.0 × 10^−54^	map00620, map00710
m.37547	K01006	pyruvate, orthophosphate dikinase	6.0 × 10^−46^	map00620, map00710
m.37548	K01006	pyruvate, orthophosphate dikinase	1.0 × 10^−54^	map00620, map00710
m.41758	K00873	pyruvate kinase	2.0 × 10^−26^	map00620, map00710
m.43095	K00873	pyruvate kinase	4.0 × 10^−31^	map00620, map00710
m.50116	K01595	phosphoenolpyruvate carboxylase	7.0 × 10^−79^	map00620, map00710
m.50118	K01595	phosphoenolpyruvate carboxylase	9.0 × 10^−40^	map00620, map00710

* The downregulated transcript;

a map00620, pyruvate metabolism; map00710, carbon fixation in photosynthetic organisms; map00190, oxidative phosphorylation.

**Table 3.** Annotations of differentially expressed transcripts involved in genetic information storage and processing.

Transcript ID	Function descriptions	*E*-value
m.56286	5′-3′ exonuclease HKE1/RAT1	9.0 × 10^−11^
m.59998	Chromatin remodeling complex SWI/SNF, component SWI2 and related ATPases (DNA/RNA helicase superfamily)	4.0 × 10^−27^
m.17815	Chromatin remodeling protein HARP/SMARCAL1, DEAD-box superfamily	8.0 × 10^−7^
m.48610	Polyadenylate-binding protein (RRM superfamily)	7.0 × 10^−6^
m.38370	Translation initiation factor 3, subunit c (eIF-3c)	3.0 × 10^−34^
m.58566	mRNA cleavage and polyadenylation factor II complex, BRR5 (CPSF subunit)	1.0 × 10^−110^
m.24433	RNA Helicase	9.0 × 10^−6^
m.12316	Transcription factor containing NAC and translation elongation factor EF-Ts, *N*-terminal domain (TS-N) domains	3.0 × 10^−8^
