# Supplementary material for: Differential Transcriptome Analysis between Paulownia fortunei and Its Synthesized Autopolyploid
Source: Int J Mol Sci. 2014 Mar 21;15(3):5079–93. doi: 10.3390/ijms15035079 (PMC3975441; doi:10.3390/ijms15035079)
Supplement: Supplementary file 1 [file ijms-15-05079-s001.pdf]

Supplementary Information

Table S1. KEGG pathways represented in *P. fortunei* transcripts.

Pathway entry	Pathway name	No. of terms	No. of transcripts
map01100	Metabolic pathways	418	1413
map01110	Biosynthesis of secondary metabolites	208	735
map01120	Microbial metabolism in diverse environments	85	394
map03013	RNA transport	54	185
map03040	Spliceosome	50	157
map04141	Protein processing in endoplasmic reticulum	46	159
map00230	Purine metabolism	45	135
map00240	Pyrimidine metabolism	39	78
map03008	Ribosome biogenesis in eukaryotes	38	141
map05169	Epstein-Barr virus infection	37	99
map04110	Cell cycle	36	114
map04111	Cell cycle—yeast	36	100
map04120	Ubiquitin mediated proteolysis	35	121
map03010	Ribosome	35	73
map03015	mRNA surveillance pathway	33	139
map04075	Plant hormone signal transduction	30	127
map00520	Amino sugar and nucleotide sugar metabolism	27	95
map04113	Meiosis—yeast	27	78
map03018	RNA degradation	26	121
map00500	Starch and sucrose metabolism	25	148
map00010	Glycolysis/Gluconeogenesis	24	156
map05016	Huntington's disease	24	74
map05166	HTLV-I infection	23	72
map04626	Plant-pathogen interaction	22	71
map03420	Nucleotide excision repair	22	62
map00620	Pyruvate metabolism	21	118

Table S1. *Cont.*

Pathway entry	Pathway name	No. of terms	No. of transcripts
map05203	Viral carcinogenesis	21	93
map04114	Oocyte meiosis	21	83
map00190	Oxidative phosphorylation	21	78
map04146	Peroxisome	21	77
map00710	Carbon fixation in photosynthetic organisms	20	111
map00970	Aminoacyl-tRNA biosynthesis	20	92
map00564	Glycerophospholipid metabolism	20	59
map00250	Alanine, aspartate and glutamate metabolism	20	57
map00270	Cysteine and methionine metabolism	19	79
map04144	Endocytosis	19	75
map04142	Lysosome	19	74
map03050	Proteasome	19	42
map03030	DNA replication	19	41
map00860	Porphyrin and chlorophyll metabolism	19	39
map05200	Pathways in cancer	18	87
map00680	Methane metabolism	18	79
map03460	Fanconi anemia pathway	18	69
map04145	Phagosome	18	62
map00330	Arginine and proline metabolism	18	57
map04151	PI3K-Akt signaling pathway	17	73
map04910	Insulin signaling pathway	16	66
map00260	Glycine, serine and threonine metabolism	16	48
map03440	Homologous recombination	15	65
map00630	Glyoxylate and dicarboxylate metabolism	15	64
map05152	Tuberculosis	15	53
map05010	Alzheimer’s disease	15	39
map00562	Inositol phosphate metabolism	14	66
map04914	Progesterone-mediated oocyte maturation	14	56
map04721	Synaptic vesicle cycle	14	54
map00510	*N*-Glycan biosynthesis	14	46
map05164	Influenza A	14	44
map03022	Basal transcription factors	14	34
map00020	Citrate cycle (TCA cycle)	13	68
map01210	2-Oxocarboxylic acid metabolism	13	60

Table S1. *Cont.*

Pathway entry	Pathway name	No. of terms	No. of transcripts
map04070	Phosphatidylinositol signaling system	13	54
map00051	Fructose and mannose metabolism	13	53
map05168	Herpes simplex infection	13	48
map00280	Valine, leucine and isoleucine degradation	13	45
map00561	Glycerolipid metabolism	13	44
map00480	Glutathione metabolism	13	43
map03430	Mismatch repair	13	41
map00900	Terpenoid backbone biosynthesis	13	36
map00195	Photosynthesis	13	29
map03410	Base excision repair	13	24
map04310	Wnt signaling pathway	12	63
map00030	Pentose phosphate pathway	12	61
map04810	Regulation of actin cytoskeleton	12	49
map00940	Phenylpropanoid biosynthesis	12	44
map00400	Phenylalanine, tyrosine and tryptophan biosynthesis	12	42
map05012	Parkinson's disease	12	31
map03020	RNA polymerase	12	24
map00052	Galactose metabolism	11	52
map00640	Propanoate metabolism	11	39
map00910	Nitrogen metabolism	11	30
map00410	beta-Alanine metabolism	11	30
map00906	Carotenoid biosynthesis	11	29
map00053	Ascorbate and aldarate metabolism	11	26
map04066	HIF-1 signaling pathway	10	56
map00720	Carbon fixation pathways in prokaryotes	10	41
map00071	Fatty acid metabolism	10	40
map05034	Alcoholism	10	34
map03060	Protein export	10	27
map05161	Hepatitis B	9	39
map04666	Fc gamma R-mediated phagocytosis	9	39
map00040	Pentose and glucuronate interconversions	9	39
map04712	Circadian rhythm—plant	9	37
map00360	Phenylalanine metabolism	9	31

Table S1. *Cont.*

Pathway entry	Pathway name	No. of terms	No. of transcripts
map05120	Epithelial cell signaling in Helicobacter pylori infection	9	30
map05110	Vibrio cholerae infection	9	30
map04115	p53 signaling pathway	9	30
map00770	Pantothenate and CoA biosynthesis	9	27
map00350	Tyrosine metabolism	9	25
map04728	Dopaminergic synapse	8	37
map00513	Various types of *N*-glycan biosynthesis	8	34
map00592	alpha-Linolenic acid metabolism	8	33
map05210	Colorectal cancer	8	30
map05323	Rheumatoid arthritis	8	29
map00600	Sphingolipid metabolism	8	26
map05134	Legionellosis	8	23
map00061	Fatty acid biosynthesis	8	19
map04722	Neurotrophin signaling pathway	8	18
map04130	SNARE interactions in vesicular transport	8	18
map00130	Ubiquinone and other terpenoid-quinone biosynthesis	8	18
map01040	Biosynthesis of unsaturated fatty acids	8	17
map00563	Glycosylphosphatidylinositol(GPI)-anchor biosynthesis	8	17
map05215	Prostate cancer	7	35
map04530	Tight junction	7	35
map04150	mTOR signaling pathway	7	32
map04966	Collecting duct acid secretion	7	27
map00650	Butanoate metabolism	7	27
map00460	Cyanoamino acid metabolism	7	26
map04612	Antigen processing and presentation	7	25
map05131	Shigellosis	7	18
map00983	Drug metabolism—other enzymes	7	15
map05202	Transcriptional misregulation in cancer	6	35
map05160	Hepatitis C	6	35
map04350	TGF-beta signaling pathway	6	35
map00920	Sulfur metabolism	6	31
map04720	Long-term potentiation	6	27

Table S1. *Cont.*

Pathway entry	Pathway name	No. of terms	No. of transcripts
map00450	Selenocompound metabolism	6	27
map04010	MAPK signaling pathway	6	26
map00670	One carbon pool by folate	6	23
map05142	Chagas disease (American trypanosomiasis)	6	22
map00290	Valine, leucine and isoleucine biosynthesis	6	22
map03320	PPAR signaling pathway	6	21
map04920	Adipocytokine signaling pathway	6	19
map00790	Folate biosynthesis	6	19
map00300	Lysine biosynthesis	6	17
map00380	Tryptophan metabolism	6	16
map00780	Biotin metabolism	6	14
map05132	Salmonella infection	6	13
map00340	Histidine metabolism	6	13
map05100	Bacterial invasion of epithelial cells	6	12
map04122	Sulfur relay system	6	11
map00196	Photosynthesis—antenna proteins	6	10
map05145	Toxoplasmosis	5	30
map04724	Glutamatergic synapse	5	29
map05020	Prion diseases	5	21
map04020	Calcium signaling pathway	5	21
map00511	Other glycan degradation	5	21
map05214	Glioma	5	19
map04710	Circadian rhythm	5	19
map05211	Renal cell carcinoma	5	18
map05130	Pathogenic Escherichia coli infection	5	18
map04510	Focal adhesion	5	18
map04391	Hippo signaling pathway—fly	5	16
map05162	Measles	5	15
map04727	GABAergic synapse	5	15
map03450	Non-homologous end-joining	5	15
map05014	Amyotrophic lateral sclerosis (ALS)	5	14
map04140	Regulation of autophagy	5	14
map04270	Vascular smooth muscle contraction	5	13
map00310	Lysine degradation	5	13

Table S1. *Cont.*

Pathway entry	Pathway name	No. of terms	No. of transcripts
map00960	Tropane, piperidine and pyridine alkaloid biosynthesis	5	10
map04623	Cytosolic DNA-sensing pathway	5	7
map00760	Nicotinate and nicotinamide metabolism	5	7
map04930	Type II diabetes mellitus	4	42
map05213	Endometrial cancer	4	22
map05212	Pancreatic cancer	4	21
map04912	GnRH signaling pathway	4	21
map04540	Gap junction	4	20
map04520	Adherens junction	4	19
map04330	Notch signaling pathway	4	19
map04730	Long-term depression	4	18
map00565	Ether lipid metabolism	4	18
map04916	Melanogenesis	4	17
map04662	B cell receptor signaling pathway	4	16
map04650	Natural killer cell mediated cytotoxicity	4	16
map04380	Osteoclast differentiation	4	16
map04370	VEGF signaling pathway	4	16
map04360	Axon guidance	4	16
map03070	Bacterial secretion system	4	15
map04210	Apoptosis	4	14
map04112	Cell cycle—Caulobacter	4	13
map00945	Stilbenoid, diarylheptanoid and gingerol biosynthesis	4	11
map00941	Flavonoid biosynthesis	4	11
map05133	Pertussis	4	10
map04620	Toll-like receptor signaling pathway	4	10
map04062	Chemokine signaling pathway	4	10
map00750	Vitamin B6 metabolism	4	9
map02020	Two-component system	4	8
map00950	Isoquinoline alkaloid biosynthesis	4	8
map00100	Steroid biosynthesis	4	8
map00904	Diterpenoid biosynthesis	4	6
map00740	Riboflavin metabolism	3	22

Table S1. *Cont.*

Pathway entry	Pathway name	No. of terms	No. of transcripts
map00521	Streptomycin biosynthesis	3	19
map05216	Thyroid cancer	3	17
map00625	Chloroalkane and chloroalkene degradation	3	17
map04723	Retrograde endocannabinoid signaling	3	16
map04621	NOD-like receptor signaling pathway	3	16
map04660	T cell receptor signaling pathway	3	15
map00830	Retinol metabolism	3	15
map00540	Lipopolysaccharide biosynthesis	3	15
map05218	Melanoma	3	14
map05140	Leishmaniasis	3	14
map00982	Drug metabolism—cytochrome P450	3	14
map00980	Metabolism of xenobiotics by cytochrome P450	3	14
map05220	Chronic myeloid leukemia	3	13
map04961	Endocrine and other factor-regulated calcium reabsorption	3	13
map00072	Synthesis and degradation of ketone bodies	3	13
map05322	Systemic lupus erythematosus	3	12
map05221	Acute myeloid leukemia	3	12
map04012	ErbB signaling pathway	3	12
map00730	Thiamine metabolism	3	12
map05031	Amphetamine addiction	3	11
map04622	RIG-I-like receptor signaling pathway	3	11
map00908	Zeatin biosynthesis	3	10
map04726	Serotonergic synapse	3	9
map04725	Cholinergic synapse	3	9
map04713	Circadian entrainment	3	9
map04664	Fc epsilon RI signaling pathway	3	9
map04064	NF-kappa B signaling pathway	3	9
map00590	Arachidonic acid metabolism	3	9
map05416	Viral myocarditis	3	8
map00531	Glycosaminoglycan degradation	3	7
map00430	Taurine and hypotaurine metabolism	3	6
map00401	Novobiocin biosynthesis	3	6
map05410	Hypertrophic cardiomyopathy (HCM)	3	5
map00905	Brassinosteroid biosynthesis	3	4

Table S1. *Cont.*

Pathway entry	Pathway name	No. of terms	No. of transcripts
map00062	Fatty acid elongation	3	4
map04260	Cardiac muscle contraction	3	3
map00591	Linoleic acid metabolism	2	23
map04614	Renin-angiotensin system	2	13
map00626	Naphthalene degradation	2	13
map04011	MAPK signaling pathway—yeast	2	12
map00603	Glycosphingolipid biosynthesis—globo series	2	11
map00660	C5-Branched dibasic acid metabolism	2	10
map00253	Tetracycline biosynthesis	2	5
map00785	Lipoic acid metabolism	2	6

Table S2. Transcripts involved in the KOG main category "information storage and processing".

Transcript ID	Log_2 fold change	KOG number	Description	KOG classification	*E* value
[J] Translation, ribosomal structure and biogenesis					
m.28330	6.25	KOG0462	Elongation factor-type GTP-binding protein	[J]	2.0×10^{-17}
m.10084	6.07	KOG1728	40S ribosomal protein S11	[J]	3.0×10^{-19}
m.57095	5.93	KOG1224	Para-aminobenzoate (PABA) synthase ABZ1	[J]	4.0×10^{-28}
m.28883	5.52	KOG1637	Threonyl-tRNA synthetase	[J]	1.0×10^{-25}
m.48494	5.46	KOG2335	tRNA-dihydrouridine synthase	[J]	3.0×10^{-7}
m.58413	4.84	KOG0328	Predicted ATP-dependent RNA helicase FAL1, involved in rRNA maturation, DEAD-box superfamily	[J]	9.0×10^{-6}
m.19713	4.75	KOG0187	40S ribosomal protein S17	[J]	1.0×10^{-7}
m.51317	4.74	KOG0433	Isoleucyl-tRNA synthetase	[J]	1.0×10^{-6}
m.37662	4.57	KOG1247	Methionyl-tRNA synthetase	[J]	2.0×10^{-12}
m.44666	4.41	KOG1033	eIF-2alpha kinase PEK/EIF2AK3	[J]	1.0×10^{-18}
m.33398	4.32	KOG2311	NAD/FAD-utilizing protein possibly involved in translation	[J]	7.0×10^{-27}
m.19718	4.19	KOG0187	40S ribosomal protein S17	[J]	1.0×10^{-7}
m.53804	3.39	KOG1041	Translation initiation factor 2C (eIF-2C) and related proteins	[J]	6.0×10^{-6}
m.9057	−2.63	KOG3203	Mitochondrial/chloroplast ribosomal protein L13	[J]	5.0×10^{-23}

Table S2. *Cont.*

Transcript ID	Log_2 fold change	KOG number	Description	KOG classification	*E* value
m.16305	−2.67	KOG0846	Mitochondrial/chloroplast ribosomal protein L15/L10	[J]	2.0×10^{-42}
m.7824	−2.86	KOG0408	Mitochondrial/chloroplast ribosomal protein S11	[J]	3.0×10^{-9}
m.16388	−3.20	KOG3184	60S ribosomal protein L7	[J]	2.0×10^{-6}
m.10083	−3.79	KOG1728	40S ribosomal protein S11	[J]	3.0×10^{-19}
m.49382	−4.35	KOG1885	Lysyl-tRNA synthetase (class II)	[J]	4.0×10^{-6}
m.33399	−4.39	KOG2311	NAD/FAD-utilizing protein possibly involved in translation	[J]	7.0×10^{-27}
m.44669	−4.44	KOG1033	eIF-2alpha kinase PEK/EIF2AK3	[J]	1.0×10^{-18}
m.26432	−4.52	KOG1191	Mitochondrial GTPase	[J]	1.0×10^{-49}
m.15618	−4.52	KOG1211	Amidases	[J]	5.0×10^{-18}
m.26182	−4.58	KOG0462	Elongation factor-type GTP-binding protein	[J]	1.0×10^{-13}
m.8736	−4.65	KOG4600	Mitochondrial ribosomal protein MRP7 (L2)	[J]	2.0×10^{-13}
m.26429	−4.68	KOG1191	Mitochondrial GTPase	[J]	3.0×10^{-51}
m.28181	−4.80	KOG0462	Elongation factor-type GTP-binding protein	[J]	3.0×10^{-19}
m.26436	−5.08	KOG1191	Mitochondrial GTPase	[J]	2.0×10^{-51}
m.34852	−5.13	KOG2509	Seryl-tRNA synthetase	[J]	1.0×10^{-15}
m.28331	−5.15	KOG0462	Elongation factor-type GTP-binding protein	[J]	2.0×10^{-17}
m.38370	−5.44	KOG1076	Translation initiation factor 3, subunit c (eIF-3c)	[J]	3.0×10^{-34}
m.15209	−5.99	KOG0875	60S ribosomal protein L5	[J]	4.0×10^{-6}
m.28882	−6.11	KOG1637	Threonyl-tRNA synthetase	[J]	1.0×10^{-25}
m.38372	−6.20	KOG1076	Translation initiation factor 3, subunit c (eIF-3c)	[J]	3.0×10^{-34}
m.50775	−6.34	KOG1145	Mitochondrial translation initiation factor 2 (IF-2; GTPase)	[J]	3.0×10^{-7}
m.10667	4.12	KOG0148	Apoptosis-promoting RNA-binding protein TIA-1/TIAR (RRM superfamily)	[AJ]	7.0×10^{-6}
m.48610	−4.71	KOG0123	Polyadenylate-binding protein (RRM superfamily)	[AJ]	7.0×10^{-6}
[A] RNA processing and modification					
m.46179	4.99	KOG2190	PolyC-binding proteins alphaCP-1 and related KH domain proteins	[AR]	1.0×10^{-6}
m.53079	4.66	KOG2190	PolyC-binding proteins alphaCP-1 and related KH domain proteins	[AR]	5.0×10^{-7}
m.46175	−4.81	KOG2190	PolyC-binding proteins alphaCP-1 and related KH domain proteins	[AR]	1.0×10^{-6}

Table S2. *Cont.*

Transcript ID	Log_2 fold change	KOG number	Description	KOG classification	*E* value
m.46178	−5.32	KOG2190	PolyC-binding proteins alphaCP-1 and related KH domain proteins	[AR]	1.0×10^{-6}
m.10667	4.12	KOG0148	Apoptosis-promoting RNA-binding protein TIA-1/TIAR (RRM superfamily)	[AJ]	7.0×10^{-6}
m.48610	−4.71	KOG0123	Polyadenylate-binding protein (RRM superfamily)	[AJ]	7.0×10^{-6}
m.57416	5.86	KOG0007	Splicing factor 3a, subunit 1	[A]	8.0×10^{-7}
m.57719	5.66	KOG1795	U5 snRNP spliceosome subunit	[A]	3.0×10^{-14}
m.48682	5.54	KOG4211	Splicing factor hnRNP-F and related RNA-binding proteins	[A]	1.0×10^{-20}
m.50750	5.50	KOG0948	Nuclear exosomal RNA helicase MTR4, DEAD-box superfamily	[A]	3.0×10^{-8}
m.59847	5.21	KOG1070	rRNA processing protein Rrp5	[A]	1.0×10^{-9}
m.28492	5.21	KOG0839	RNA Methylase, SpoU family	[A]	2.0×10^{-53}
m.38309	5.10	KOG2636	Splicing factor 3a, subunit 3	[A]	2.0×10^{-10}
m.29695	5.09	KOG0335	ATP-dependent RNA helicase	[A]	3.0×10^{-6}
m.56203	5.02	KOG2098	Predicted N6-adenine RNA methylase	[A]	2.0×10^{-10}
m.14486	4.98	KOG1801	tRNA-splicing endonuclease positive effector (SEN1)	[A]	6.0×10^{-6}
m.47208	4.88	KOG0120	Splicing factor U2AF, large subunit (RRM superfamily)	[A]	2.0×10^{-6}
m.53303	4.83	KOG1898	Splicing factor 3b, subunit 3	[A]	1.0×10^{-59}
m.57469	4.82	KOG0951	RNA helicase BRR2, DEAD-box superfamily	[A]	4.0×10^{-11}
m.53500	4.63	KOG1960	Predicted RNA-binding protein, contains KH domains	[A]	4.0×10^{-7}
m.14915	4.52	KOG0953	Mitochondrial RNA helicase SUV3, DEAD-box superfamily	[A]	4.0×10^{-32}
m.29694	4.51	KOG0335	ATP-dependent RNA helicase	[A]	3.0×10^{-6}
m.58671	4.42	KOG0701	dsRNA-specific nuclease Dicer and related ribonucleases	[A]	1.0×10^{-6}
m.58664	4.36	KOG0701	dsRNA-specific nuclease Dicer and related ribonucleases	[A]	5.0×10^{-8}
m.57190	4.33	KOG0920	ATP-dependent RNA helicase A	[A]	3.0×10^{-35}
m.21079	4.29	KOG0106	Alternative splicing factor SRp55/B52/SRp75 (RRM superfamily)	[A]	1.0×10^{-7}
m.51740	4.20	KOG0922	DEAH-box RNA helicase	[A]	1.0×10^{-9}
m.10895	4.09	KOG4768	Mitochondrial mRNA maturase	[A]	1.0×10^{-9}
m.50245	3.35	KOG1895	mRNA cleavage and polyadenylation factor II complex, subunit PTA1	[A]	3.0×10^{-12}

Table S2. *Cont.*

Transcript ID	Log₂ fold change	KOG number	Description	KOG classification	*E* value
m.12812	2.78	KOG2202	U2 snRNP splicing factor, small subunit, and related proteins	[A]	4.0×10^{-9}
m.48327	−4.20	KOG0331	ATP-dependent RNA helicase	[A]	4.0×10^{-6}
m.44747	−4.29	KOG0920	ATP-dependent RNA helicase A	[A]	2.0×10^{-6}
m.44746	−4.33	KOG0920	ATP-dependent RNA helicase A	[A]	2.0×10^{-6}
m.58224	−4.39	KOG0988	RNA-directed RNA polymerase QDE-1 required for posttranscriptional gene silencing and RNA interference	[A]	5.0×10^{-6}
m.47203	−4.43	KOG0120	Splicing factor U2AF, large subunit (RRM superfamily)	[A]	8.0×10^{-8}
m.48680	−4.44	KOG4211	Splicing factor hnRNP-F and related RNA-binding proteins	[A]	1.0×10^{-20}
m.15544	−4.51	KOG4205	RNA-binding protein musashi/mRNA cleavage and polyadenylation factor I complex, subunit HRP1	[A]	1.0×10^{-5}
m.14916	−4.52	KOG0953	Mitochondrial RNA helicase SUV3, DEAD-box superfamily	[A]	4.0×10^{-32}
m.10894	−4.64	KOG4768	Mitochondrial mRNA maturase	[A]	1.0×10^{-9}
m.38075	−4.74	KOG0108	mRNA cleavage and polyadenylation factor I complex, subunit RNA15	[A]	9.0×10^{-6}
m.57417	−4.81	KOG0007	Splicing factor 3a, subunit 1	[A]	8.0×10^{-7}
m.58566	−5.02	KOG1137	mRNA cleavage and polyadenylation factor II complex, BRR5 (CPSF subunit)	[A]	1.0×10^{-110}
m.38310	−5.36	KOG2636	Splicing factor 3a, subunit 3	[A]	2.0×10^{-10}
m.24433	−5.54	KOG0343	RNA Helicase	[A]	9.0×10^{-6}
m.59844	−5.66	KOG1070	rRNA processing protein Rrp5	[A]	1.0×10^{-9}
m.28491	−6.00	KOG0839	RNA Methylase, SpoU family	[A]	4.0×10^{-62}
m.28471	4.75	KOG2044	5'-3' exonuclease HKE1/RAT1	[LA]	9.0×10^{-8}
m.56286	−4.20	KOG2044	5'-3' exonuclease HKE1/RAT1	[LA]	9.0×10^{-11}
m.25310	4.03	KOG1902	Putative signal transduction protein involved in RNA splicing	[TA]	3.0×10^{-8}
[K] Transcription					
m.57710	5.59	KOG1513	Nuclear helicase MOP-3/SNO (DEAD-box superfamily)	[KT]	1.0×10^{-81}
m.57714	5.35	KOG1513	Nuclear helicase MOP-3/SNO (DEAD-box superfamily)	[KT]	2.0×10^{-7}
m.57712	5.00	KOG1513	Nuclear helicase MOP-3/SNO (DEAD-box superfamily)	[KT]	2.0×10^{-81}
m.57385	6.64	KOG1831	Negative regulator of transcription	[K]	9.0×10^{-31}

Table S2. *Cont.*

Transcript ID	Log2 fold change	KOG number	Description	KOG classification	*E* value
m.57769	5.13	KOG1952	Transcription factor NF-X1, contains NFX-type Zn^{2+}-binding and R3H domains	[K]	2.0×10^{-7}
m.56319	4.99	KOG0048	Transcription factor, Myb superfamily	[K]	3.0×10^{-6}
m.16149	4.89	KOG3227	Calcium-responsive transcription coactivator	[K]	1.0×10^{-6}
m.14891	4.58	KOG0484	Transcription factor PHOX2/ARIX, contains HOX domain	[K]	9.0×10^{-6}
m.60202	4.51	KOG4522	RNA polymerase II transcription mediator	[K]	4.0×10^{-25}
m.32248	4.39	KOG0048	Transcription factor, Myb superfamily	[K]	1.0×10^{-5}
m.59358	4.36	KOG1778	CREB binding protein/P300 and related TAZ Zn-finger proteins	[K]	5.0×10^{-15}
m.22814	4.25	KOG1601	GATA-4/5/6 transcription factors	[K]	6.0×10^{-6}
m.34174	4.20	KOG1598	Transcription initiation factor TFIIIB, Brf1 subunit	[K]	5.0×10^{-28}
m.57453	4.16	KOG1356	Putative transcription factor 5qNCA, contains JmjC domain	[K]	6.0×10^{-11}
m.53446	4.15	KOG1913	Regucalcin gene promoter region-related protein (RGPR)	[K]	8.0×10^{-29}
m.22815	2.78	KOG1601	GATA-4/5/6 transcription factors	[K]	9.0×10^{-6}
m.20168	2.47	KOG1601	GATA-4/5/6 transcription factors	[K]	2.0×10^{-7}
m.12316	−3.40	KOG2239	Transcription factor containing NAC and TS-N domains	[K]	3.0×10^{-8}
m.56318	−4.20	KOG0048	Transcription factor, Myb superfamily	[K]	3.0×10^{-6}
m.43112	−4.22	KOG0528	HMG-box transcription factor SOX5	[K]	9.0×10^{-6}
m.57770	−4.30	KOG1952	Transcription factor NF-X1, contains NFX-type Zn2+-binding and R3H domains	[K]	2.0×10^{-7}
m.26017	−4.58	KOG1601	GATA-4/5/6 transcription factors	[K]	1.0×10^{-9}
m.2754	−4.70	KOG1601	GATA-4/5/6 transcription factors	[K]	9.0×10^{-6}
m.9910	−4.89	KOG1668	Elongation factor 1 beta/delta chain	[K]	4.0×10^{-7}
m.40477	−5.00	KOG2039	Transcriptional coactivator p100	[K]	5.0×10^{-34}
m.9912	−5.09	KOG1668	Elongation factor 1 beta/delta chain	[K]	4.0×10^{-7}
m.22067	−5.21	KOG1778	CREB binding protein/P300 and related TAZ Zn-finger proteins	[K]	4.0×10^{-6}
m.26018	−5.33	KOG1601	GATA-4/5/6 transcription factors	[K]	1.0×10^{-9}

Table S2. *Cont.*

Transcript ID	Log_2 fold change	KOG number	Description	KOG classification	*E* value
m.58254	5.21	KOG0973	Histone transcription regulator HIRA, WD repeat superfamily	[DK]	2.0×10^{-8}
m.30185	6.90	KOG2633	Hismacro and SEC14 domain-containing proteins	[BK]	4.0×10^{-7}
m.42532	4.86	KOG0386	Chromatin remodeling complex SWI/SNF, component SWI2 and related ATPases (DNA/RNA helicase superfamily)	[BK]	1.0×10^{-12}
m.22726	4.52	KOG2626	Histone H3 (Lys4) methyltransferase complex, subunit CPS60/ASH2/BRE2	[BK]	5.0×10^{-9}
m.59998	−4.45	KOG0386	Chromatin remodeling complex SWI/SNF, component SWI2 and related ATPases (DNA/RNA helicase superfamily)	[BK]	4.0×10^{-27}
[L] Replication, recombination and repair					
m.27619	−4.90	KOG2045	5'-3' exonuclease XRN1/KEM1/SEP1 involved in DNA strand exchange and mRNA turnover	[LD]	2.0×10^{-9}
m.28471	4.75	KOG2044	5'-3' exonuclease HKE1/RAT1	[LA]	9.0×10^{-8}
m.56286	−4.20	KOG2044	5'-3' exonuclease HKE1/RAT1	[LA]	9.0×10^{-11}
m.17741	6.22	KOG1508	DNA replication factor/protein phosphatase inhibitor SET/SPR-2	[L]	2.0×10^{-6}
m.60241	5.37	KOG0891	DNA-dependent protein kinase	[L]	2.0×10^{-6}
m.22798	5.13	KOG0217	Mismatch repair ATPase MSH6 (MutS family)	[L]	1.0×10^{-6}
m.40148	5.04	KOG2179	Nucleotide excision repair complex XPC-HR23B, subunit XPC/DPB11	[L]	2.0×10^{-28}
m.41495	4.86	KOG1978	DNA mismatch repair protein—MLH2/PMS1/Pms2 family	[L]	9.0×10^{-6}
m.48621	4.62	KOG1956	DNA topoisomerase III alpha	[L]	2.0×10^{-13}
m.38736	4.52	KOG2310	DNA repair exonuclease MRE11	[L]	7.0×10^{-12}
m.41493	4.38	KOG1978	DNA mismatch repair protein—MLH2/PMS1/Pms2 family	[L]	9.0×10^{-6}
m.8216	4.24	KOG0221	Mismatch repair ATPase MSH5 (MutS family)	[L]	9.0×10^{-6}
m.49196	4.17	KOG0250	DNA repair protein RAD18 (SMC family protein)	[L]	5.0×10^{-6}
m.18130	4.09	KOG1132	Helicase of the DEAD superfamily	[L]	4.0×10^{-32}
m.41492	−4.53	KOG1978	DNA mismatch repair protein—MLH2/PMS1/Pms2 family	[L]	9.0×10^{-6}

Table S2. *Cont.*

Transcript ID	Log_2 fold change	KOG number	Description	KOG classification	*E* value
m.49197	−4.75	KOG0250	DNA repair protein RAD18 (SMC family protein)	[L]	5.0×10^{-6}
m.60242	−5.02	KOG0891	DNA-dependent protein kinase	[L]	2.0×10^{-6}
[B] Chromatin structure and dynamics					
m.30185	6.90	KOG2633	Hismacro and SEC14 domain-containing proteins	[BK]	4.0×10^{-7}
m.42532	4.86	KOG0386	Chromatin remodeling complex SWI/SNF, component SWI2 and related ATPases (DNA/RNA helicase superfamily)	[BK]	1.0×10^{-12}
m.22726	4.52	KOG2626	Histone H3 (Lys4) methyltransferase complex, subunit CPS60/ASH2/BRE2	[BK]	5.0×10^{-9}
m.59998	−4.45	KOG0386	Chromatin remodeling complex SWI/SNF, component SWI2 and related ATPases (DNA/RNA helicase superfamily)	[BK]	4.0×10^{-27}
m.35864	4.48	KOG2266	Chromatin-associated protein Dek and related proteins, contains SAP DNA binding domain	[B]	4.0×10^{-9}
m.48936	4.40	KOG1279	Chromatin remodeling factor subunit and related transcription factors	[B]	2.0×10^{-14}
m.59774	4.09	KOG0389	SNF2 family DNA-dependent ATPase	[B]	7.0×10^{-6}
m.17815	−4.21	KOG1000	Chromatin remodeling protein HARP/SMARCAL1, DEAD-box superfamily	[B]	8.0×10^{-7}

Table S3. Primer sequences used for verification of differentially expressed transcripts between diploid and autotetraploid *P. fortunei.*

Transcript ID	5'-primer sequence	3'-primer sequence
m.30899	TGGCTCCTCTCCTTACTATG	AACAATCTGCGGCAATCC
m.33871	TCAGGCACTCTCCAAGTATTC	CGGCATATTTGAGGTGTTAGC
m.54501	GCCAATGACCCAACAATAGAAC	CGAAGAGCATCAGCATAAGAAC
m.32555	ACCTGGGCAAGCAATCTC	TCCACGGTGAACAAGTCC
m.14097	GGGCTTGCTGCTGGAATG	CCAACAATAAGACCATACAGTGC
m.28729	AAAGAGGGTATGGAAAGGAAAGAC	ATTGGTGTTGGCAGGATTAGC
m.8309	GGTGTTGGTGGTGGTGTTG	AAGTCCTTTGTTGTGGTGTGG
m.32221	ATGACCCTGTTAGCAATCTCTTAG	GCCTCAACCACCTCCTTCG
m.41758	ATTCTAATGCCATCGCTACTAC	CGTGCCACATTCATTCCAG
m.43095	AGCATTCGGAGGAGCCAAG	GCGGAGTAGGTTCGGTGAC
m.37547	CTATGCCTGGAATGATGG	AAGTGAATGTGGAATGCC
m.37548	GAGAGGATTAAGGCTGTGAG	GGTCTAGGAGTCTGATTGTC
m.50116	TGATGGTTGGATATTCTGACTC	GATTGAATGGCAAGATATGTAGG
m.50118	TCAACAGAGCAAGGAGAAATGG	GCCGTAAGGTAGCAAGAAGC
m.56286	CCACCAGAAGGAGTTACAATG	GGTAAATCTGCTCTGAACAATAG
m.59998	AGGAAGAAGAAAGGAACCGTATAG	GCCAAGCCTGAACACCATC
m.17815	GCATTTCTCTTGAGTAGC	CTCTTCTTCTGTCCATCC
m.48610	GAGGAGAATGGAGTGGCAGAG	AAGCGAAGATGGGAGTGTGG
m.38370	CGTCAGATGCCTTACCAC	CCGCTCACTCACTTCAAG
m.58566	TCACCAGGAAGGACACTAAG	CAAGGACATACCAACAAATACC
m.24433	ATGAAGTGCTCCAAGTTG	GCCTGCTGAATAACATCG
m.12316	AGACACAGGCTGCTCAAC	CATCAACCTCCTCCTCTTCC

Figure S1. Gene Ontology Classification for complete transcripts of *P. fortunei* transcriptome.

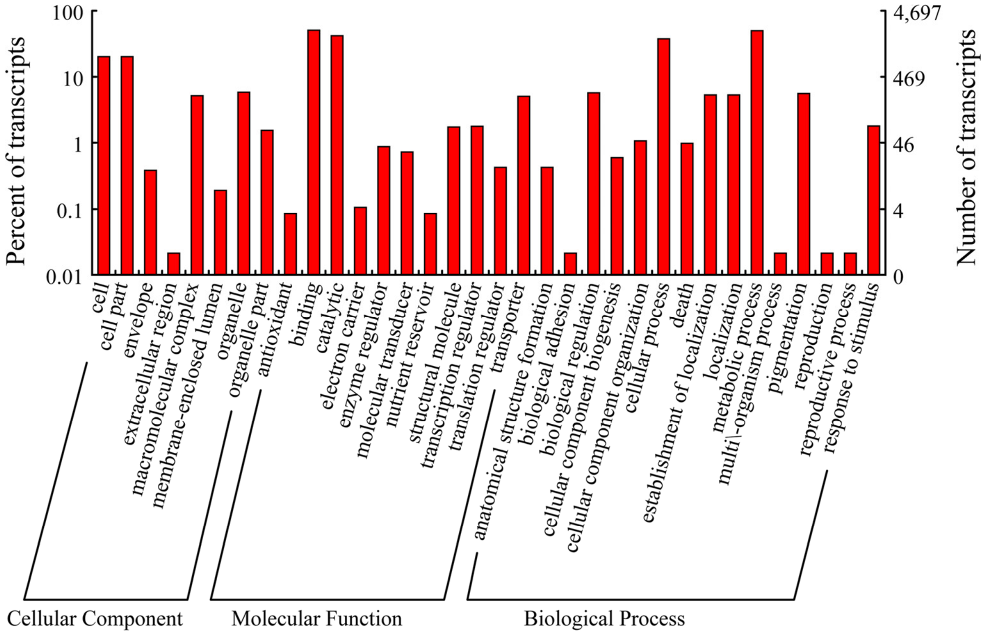

Figure S2. Histogram presentation of KOG classification for complete transcripts of *P. fortunei* transcriptome.

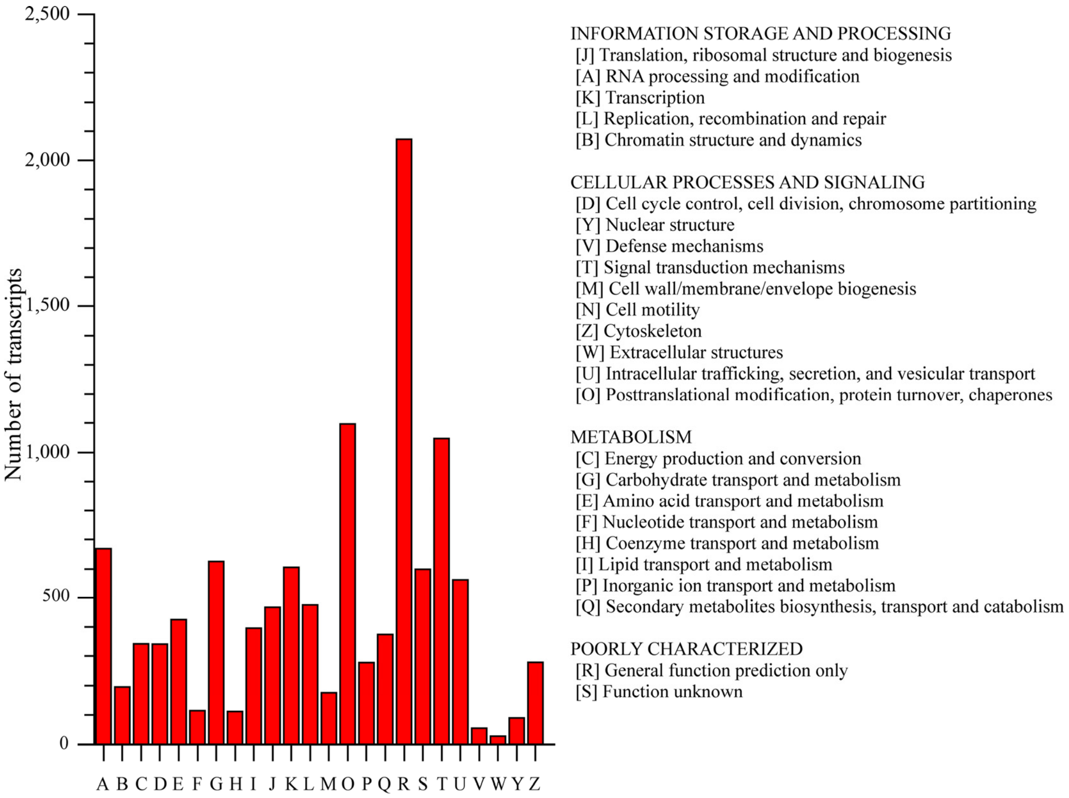

Figure S3. KOG functional categorization of differentially expressed transcripts of *P. fortunei* transcriptome.

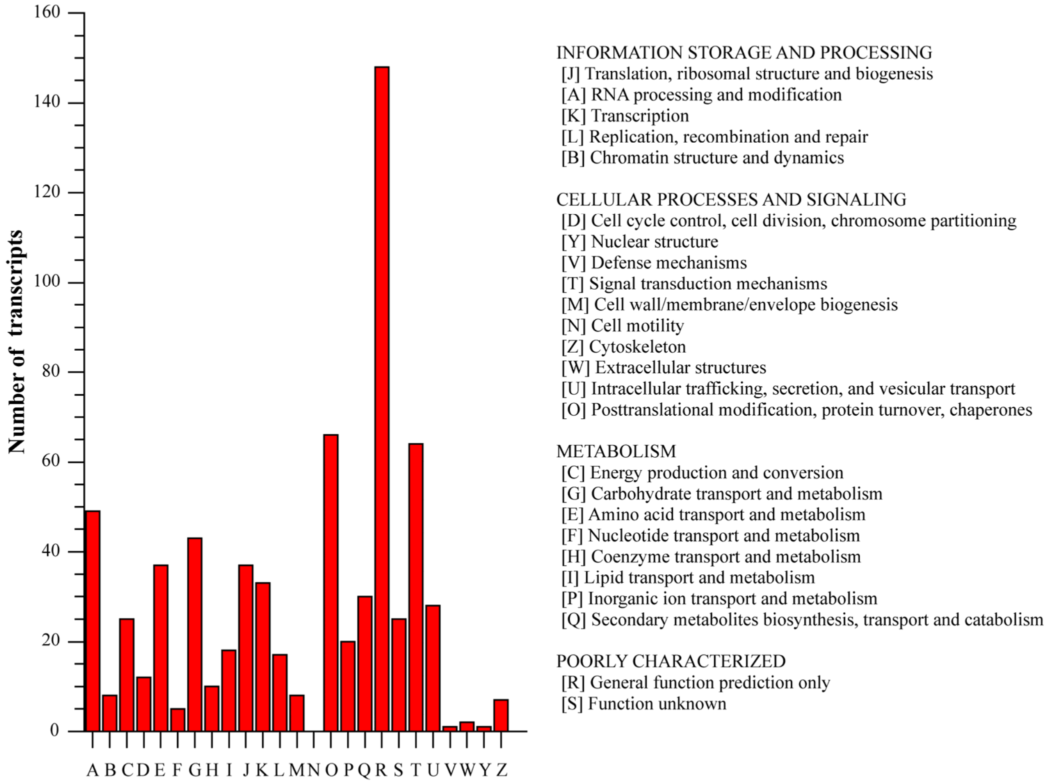

1. The options of Bowtie used in this study

The command line: bowtie2 -x ref.fasta -1 1.fq -2 2.fq -S output.sam

Options:
Input:
-q
-s/--skip <none>
-u/--upto <no limit>
-5/--trim5 <0>
-3/--trim3 <0>
--phred33
Presets: Same as:
For --end-to-end:
--sensitive -D 15 -R 2 -N 0 -L 22 -i S,1,1.15
Alignment:
-N <0>
-L <22>
-i <S,1,1.15>
--n-ceil <L,0,0.15>
--dpad <15>

--gbar <4>
--ignore-quals off
--nofw off
--norc off
--end-to-end on
--local off
Scoring:
--ma <0>
--mp <6>
--np <1>
--rdg <5>, <3>
--rfg <5>, <3>
--score-min <L,-0.6,-0.6>
Reporting: (default)
Effort:
-D <15>
-R <2>
Paired-end:
-I/--minins <0>
-X/--maxins <500>
--fr
--no-mixed
--no-discordant
--no-dovetail
--no-contain
--no-overlap
Output:
-t/--time
--quiet
--met-file <path> off
--met-stderr off
--met <1>
--no-head
--no-sq
--rg-id <text>
--rg <text>
--omit-sec-seq
Performance:
-o/--offrate <int>
-p/--threads <1>
--reorder
--mm

Other:
--qc-filter
--seed <0>
--non-deterministic
--version
-h/--help

2. The options of RSEM used in this study

The command line: perl run_RSEM_align_n_estimate.pl --transcripts ref.fasta --seqType fq --left 1.fq --right 2.fq

Options:
--prefix <RSEM>
--SS_lib_type <string> FR
--no_group_by_component
--thread_count 4
--debug

© 2014 by the authors; licensee MDPI, Basel, Switzerland. This article is an open access article distributed under the terms and conditions of the Creative Commons Attribution license (http://creativecommons.org/licenses/by/3.0/).
